# Effects of Sulforaphane and 3,3′-Diindolylmethane on Genome-Wide Promoter Methylation in Normal Prostate Epithelial Cells and Prostate Cancer Cells

**DOI:** 10.1371/journal.pone.0086787

**Published:** 2014-01-22

**Authors:** Carmen P. Wong, Anna Hsu, Alex Buchanan, Zoraya Palomera-Sanchez, Laura M. Beaver, E. Andres Houseman, David E. Williams, Roderick H. Dashwood, Emily Ho

**Affiliations:** 1 School of Biological & Population Health Sciences, Oregon State University, Corvallis, Oregon, United States of America; 2 Linus Pauling Institute, Oregon State University, Corvallis, Oregon, United States of America; 3 Department of Environmental and Molecular Toxicology, Oregon State University, Corvallis, Oregon, United States of America; 4 Moore Family Center for Whole Grain Foods, Nutrition and Preventive Health, Oregon State University, Corvallis, Oregon, United States of America; The University of Arizona, United States of America

## Abstract

Epigenetic changes, including aberrant DNA methylation, result in altered gene expression and play an important role in carcinogenesis. Phytochemicals such as sulforaphane (SFN) and 3,3′-diindolylmethane (DIM) are promising chemopreventive agents for the treatment of prostate cancer. Both have been shown to induce re-expression of genes, including tumor suppressor genes silenced in cancer cells, via modulation of epigenetic marks including DNA methylation. However, it remained unclear the effects SFN and DIM on DNA methylation at a genomic scale. The goal of this study was to determine the genome-wide effects of SFN and DIM on promoter methylation in normal prostate epithelial cells and prostate cancer cells. Both SFN and DIM treatment decreased DNA methyltransferase expression in normal prostate epithelial cells (PrEC), and androgen-dependent (LnCAP) and androgen-independent (PC3) prostate cancer cells. The effects of SFN and DIM on promoter methylation profiles in normal PrEC, LnCAP and PC3 prostate cancer cells were determined using methyl-DNA immunoprecipitation followed by genome-wide DNA methylation array. We showed widespread changes in promoter methylation patterns, including both increased and decreased methylation, in all three prostate cell lines in response to SFN or DIM treatments. In particular, SFN and DIM altered promoter methylation in distinct sets of genes in PrEC, LnCAP, and PC3 cells, but shared similar gene targets within a single cell line. We further showed that SFN and DIM reversed many of the cancer-associated methylation alterations, including aberrantly methylated genes that are dysregulated or are highly involved in cancer progression. Overall, our data suggested that both SFN and DIM are epigenetic modulators that have broad and complex effects on DNA methylation profiles in both normal and cancerous prostate epithelial cells. Results from our study may provide new insights into the epigenetic mechanisms by which SFN and DIM exert their cancer chemopreventive effects.

## Introduction

Epigenetic mechanisms are essential for regulating and maintaining gene expression patterns. Dysregulated epigenetic processes, including aberrant DNA methylation, histone modification, and microRNA profiles, lead to altered gene expression and function and play an important role in carcinogenesis. In particular, widespread changes in DNA methylation patterns are observed during cancer initiation and progression, characterized by global and site-specific DNA hypomethylation, as well as gene-specific promoter hypermethylation [1], [2]. DNA hypomethylation in cancer can contribute to genome instability and increased expression of oncogenes. On the other hand, DNA hypermethylation can lead to silencing of tumor suppressor genes, transcription factors, as well as genes involved in cell cycle regulation and apoptosis. The establishment and maintenance of DNA methylation patterns are mediated by DNA methyltransferases (DNMTs) [3]. Overexpression of DNMTs is observed in many cancers, including leukemia [4], pancreatic cancer [5], gastric cancer [6], lung cancer [7], and prostate cancer [8], and dysregulated DNMT expression likely is one of the contributing factors leading to aberrant DNA methylation patterns during cancer progression. Unlike genetic mutations, epigenetic alterations are potentially reversible and represent an attractive and promising target for cancer chemoprevention strategies. Many epigenetic drugs developed to reverse DNA methylation and histone modification aberrations in cancer are currently under investigation. In addition to pharmacologic agents, an increasing number of essential micronutrients and dietary phytochemicals have been shown to act as epigenetics modulators, and are attractive candidates for use in epigenetic therapy [9], [10]. The ability of dietary factors to exert epigenetic effects underscores the potential importance of specific nutrients and bioactive phytochemicals in epigenetic regulation and cancer chemoprevention strategies.

Prostate cancer is the second most common diagnosed cancer in men in the United States [11]. Diet is a modifiable risk factor and can influence the susceptibility to prostate cancer development. Prostate cancer risk has been shown to be inversely correlated with the consumption of cruciferous vegetables [12], [13]. In particular, sulforaphane (SFN) and 3,3′-diindolylmethane (DIM), two phytochemicals derived from glucosinolates in cruciferous vegetables have been demonstrated to be effective chemopreventive agents against prostate cancer [14], [15]. SFN is an isothiocyanate derived from the hydrolysis of glucoraphanin, and DIM is a major acid condensation product of indole-3-carbinol (I3C), a hydrolysis product of glucobrassicin. The anti-cancer effects of both SFN and DIM are multi-faceted, involving various chemopreventive mechanisms including the induction of Phase 2 enzymes, increase in apoptosis, induction of cell cycle arrest, and inhibition of cell proliferation. More recently, increasing evidence indicates that SFN and DIM may also act as epigenetics modulators and exert anti-cancer properties by targeting epigenetic marks in prostate cancer cells. For example, SFN and DIM can inhibit histone deacetylase (HDAC) activities, alter HDAC expression, and result in re-expression of tumor suppressor genes [16], [17]. We and others have shown that SFN can inhibit DNMT expression and alter DNA methylation in prostate and breast cancer cells, representing a novel chemoprevention mechanism by which SFN epigenetically regulates gene expression [18]–[20]. The dual effects of SFN on HDAC inhibition and DNA methylation make it an attractive dietary chemoprevention agent. However, little is known regarding the effects of SFN on other aberrantly methylated gene targets, and whether SFN have differential effects on DNA methylation in normal and cancerous prostate cells. In addition, it remains to be determined if DIM can similarly exert dual epigenetic effects and modulate DNA methylation in prostate cancer cells.

This current study was undertaken to determine the genome-wide effects of SFN and DIM on promoter methylation in normal prostate epithelial cells and prostate cancer cells. We hypothesize that both SFN and DIM are effective dietary modulators of DNA methylation due to their inhibitory effects on DNMT expression. We further hypothesize that SFN and DIM can differentially affect the promoter methylation profiles in normal and cancerous prostate epithelial cells, and reverse aberrant methylated genes in prostate cancer cells. Our study will increase our understanding of the methylation targets of SFN and DIM, and provide insights into the epigenetic mechanisms by which SFN and DIM exert their cancer chemopreventive effects.

## Materials and Methods

### Cell Culture and Treatment Conditions

Normal human prostate epithelial cells (PrEC) were obtained from Lonza (Allendale, NJ) and cultured in PrEC basal media containing PrEGM SingleQuot Kit supplements and growth factors (Lonza, Allendale, NJ). Human androgen-dependent prostate cancer epithelial cells (LnCAP) and androgen-independent prostate cancer epithelial cells (PC3) were obtained from American Type Culture Collection (Manassas, VA), and cultured in RPMI1640 media supplemented with 10% fetal bovine serum. All cells were cultured in humidified incubator at 5% CO_2_ and 37°C. SFN (LKT Laboratories, St. Paul, MN) and DIM (Sigma, St. Louis, MO) were dissolved in dimethylsulfoxide (DMSO). PrEC, LnCAP, and PC3 cells were treated with vehicle control (0.03% DMSO), 15 µM SFN, or 15 µM DIM in biological triplicates for use in DNA methylation arrays. The treatment dose was chosen to reflect physiologically relevant concentrations of SFN and DIM [21], [22]. Cells were collected at 48 h post-treatments for use in methylation assays or Chromatin Immunoprecipitation (ChIP) assays. For gene expression assays, cells were collected at 72 h post-treatment. In groups treated with DNA demethylating agent, 5-aza-2′-deoxycytidine (AZA), 5 µM AZA were used and cells were collected at 48 h post-treatment.

### Sample Preparation for DNA Methylation Array

A total of 27 samples were submitted for DNA methylation array analysis, including samples from each of the three cell lines treated with vehicle controls (n = 3 per cell line), DIM (n = 3 per cell line), and SFN (n = 3 per cell line). Genomic DNA was isolated using DNeasy Blood & Tissue Kit (Qiagen, Valencia, CA). For genomic DNA fragmentation, purified DNA was digested with MseI restriction enzyme (New England Biolabs, Ipswich, MA) overnight at 37°C to yield DNA fragments between 200 to 1,000 bp. Methylated DNA was enriched using methyl-DNA immunoprecipitation (MeDIP) according to manufacturer’s protocol (Roche NimbleGen, Madison, WI). MeDIP-enriched DNA as well as input DNA was amplified with GenomePlex Complete Whole Genome Amplification kit (Sigma). Amplified MeDIP and input DNA samples were submitted to Center for Genome Research & Biocomputing core facility (Oregon State University, Corvallis, OR) for sample labeling, sample hybridization, and array scanning. Sample hybridization and array scanning were done using NimbleGen Hybridization System 4 (Roche) and Axon GenePix Pro 4200 A (Molecular Device, Sunnyvale, CA), respectively.

### DNA Methylation Array Data Analysis

NimbleGen Human DNA Methylation 3×720 K CpG Island Plus RefSeq Promoter Array (Roche) based on the HG18 genome release was used. The array contained 720,000 probes of 50–75 bp in length with a median probe spacing of 104 bp, covering 30,848 transcripts, 22,532 promoters, and 27,728 CpG islands. The raw intensities of the scanned image for both Cy5 and Cy3 channels were extracted using the NimbleScan (Roche). The raw intensity pair files were imported into the R statistical programming environment using custom R software.

#### Identification of probes with significant scaled log_2_ ratio

The signal intensity ratios, were generated by subtracting the log transformed IP channel intensities from the log transformed Input channel intensities. The ratios were centered on a per sample basis by the Tukey biweight function. Probes with significant scaled log_2_ ratio were identified by NimbleScan software using default parameters as provided by the manufacturer.

#### Differential methylation fold-change between cell lines and/or treatments

Pairwise comparisons were performed through re-parameterization to determine cell line and treatment effects. Standard errors and degrees-of-freedom were extracted and used for constructing t-statistics and determination of significance.

Methylation array data were deposited into NCBI Gene Expression Omnibus, accession number GSE47017.

A list of probes with significant log2 fold-change (p-value <0.05) comparing between treatment (SFN or DIM) versus vehicle control, or comparing between prostate cancer cells (vehicle control) versus normal prostate epithelial cells (vehicle control) were generated. Probes with significant fold-change were mapped to annotated features in the human genome (HG18) and visualized using Generic Genome Browser (GBrowse) [23]. Probes within 2 kb upstream and 1 kb downstream of transcription start site (TSS) were included in the analyses in this report. Hierarchical clustering analyses were done using MeV software [24]. Venn diagrams comparing the overlap of different gene lists were generated with BioVenn [25]. Gene enrichment and functional annotation analyses were done using Database for Annotation, Visualization and Integrated Discovery v6.7 (DAVID) [26]. Functional annotation clustering tool was used to determine significantly enriched Gene Ontology (GO) terms within each annotation clusters. Epigenome mapping of significantly differentially methylated probes to ENCODE histone modification database was done using EpiExplorer [27].

### Validation of DNA Methylation Data

Select differentially methylated genes as determined by NimbleGen methylation array were validated by pyrosequencing. Genomic DNA was treated with sodium bisulfite using EpiTech Bisulfite Kit (Qiagen). Pyrosequencing PCR and sequencing primers for select differentially methylated genes were designed using PyroMark Assay Design version 2.0.1 software (Qiagen) (Table S1). PCR amplification of bisulfite-converted genomic DNA was done using PyroMark PCR kit (Qiagen) under conditions as specified by the manufacturer. PCR products were submitted to Stanford University Protein and Nucleic Acid Facility (Palo Alto, CA) for pyrosequencing. Quantitative methylation analyses were done using PyroMark Q23 version 2.0.6 software (Qiagen).

### Gene Expression Analyses

Total RNA from treated cells was isolated using Trizol (Life Technologies). Total RNA was reverse transcribed into cDNA using SuperScript III First-Strand Synthesis SuperMix for qRT-PCR (Life Technologies). Gene expression was quantified by qPCR using the following qPCR primers: human DNMT1 (forward: 5′-GTGGGGGACTGTGTCTCTGT-3′, reverse: 5′-TGAAAGCTGCATGTCCTCAC-3′), DNMT3A (forward: 5′-CACACAGAAGCATATCCAGGAGTG-3′, reverse: 5′-AGTGGACTGGGAAACCAAATACCC-3′), DNMT3B (forward: 5′-AATGTGAATCCAGCCAGGAAAGGC-3′, reverse: 5′-ACTGGATTACACTCCAGGAACCGT-3′), GAPDH (forward: 5′-CGAGATCCCTCCAAAATCAA-3′, reverse: 5′-TTCACACCCATGACGAACAT-3′), CCR4 (forward: 5′-AATTGTGCACGCGGTGTTTT-3′, reverse: 5′-TCCAGGGAGCTGAGAACCTT-3′), CXCR4 (forward: 5′-GAACTTCCTATGCAAGGCAGTCC-3′, reverse: 5′- CCATGATGTGCTGAAACTGGAAC-3′), CYR61 (forward: 5′-CTTAGTCGTCACCCTTCTCCAC-3′, reverse: 5′-CAGGGTCTGCCCTCTGACT-3′), and TGFBR1 (forward: 5′-CCTCGAGATAGGCCGTTTGT-3′, reverse: 5′-ATGGTGAATGACAGTGCGGT-3′). All qPCR reactions were done using Fast SYBR Green Mastermix (Life Technologies) on 7900 HT Fast Real-Time PCR System (Applied Biosystems). Gene expression was normalized to GAPDH and relative quantification was determined using the ΔΔCt method in RQ Manager 1.2.1 software (Applied Biosystems).

### Chromatin Immunoprecipitation (ChIP) Analyses

ChIP analyses were done as previously described with slight modifications [28]. Briefly, treated cells were fixed in formaldehyde and chromatin was sheared by sonication. Protein/DNA complexes were immunoprecipitated with antibodies specific against H3K4me3 (Abcam, Cambridge, MA). Negative control ChIP was done using normal IgG. Following immunoprecipitation, cross-linking reversal, and proteinase K treatment, ChIP DNA was purified by phenol-chloroform extraction. ChIP-qPCR primers were designed to amplify specific TGFBR1 and CYR61 promoter regions that showed differential methylation due to DIM treatment (TGFBR1 forward: 5′-GGATCGGGAAGGGGTTTGAG-3′, reverse: 5′-CCCTTCACATGCGACTCACT-3′; CYR61 forward: 5′-CTCCCACCCCTAACCCTCTA-3′, reverse: 5′-GGCCCTTAGTGCTAATGCTGA-3′). ChIP-qPCR reactions were done in triplicate, and quantitation of immunoprecipitated DNA samples was done using a standard curve generated from serial dilutions of purified input DNA. Results were calculated as a percentage of input DNA (% input) and reported as relative fold-change compared to DMSO vehicle control.

### Statistical Analysis

Statistical testing of EpiExplorer overlap values to determine whether select methylation probes overlap significantly more than expected by chance (randomized control) with H3K4me3 and H3K9ac peaks in the ENCODE data base were done using Sequential Monte Carlo multiple testing (MCFDR) algorithm in Genomic HyperBrowser [29], [30]. Statistical difference between DNA probes associated with DIM-mediated increased or decreased methylation and their respective overlap with histone marks were determined by the two-sample proportion t-test in SciPy (http://scipy.org). Remaining statistical analyses were performed using GraphPad Prism Version 5.02(GraphPad, La Jolla, CA), where data were reported as mean ± SEM, and p-values were determined using unpaired t test or one-way ANOVA follow by Tukey-Krammer Multiple Comparison test where appropriate. Statistical level of significance was defined as α of 0.05.

## Results

### SFN and DIM Decreased DNMT Gene Expression and caused Distinct DNA Methylation Profile Alterations Depending on Prostate Cell Line

**Figure 1:**
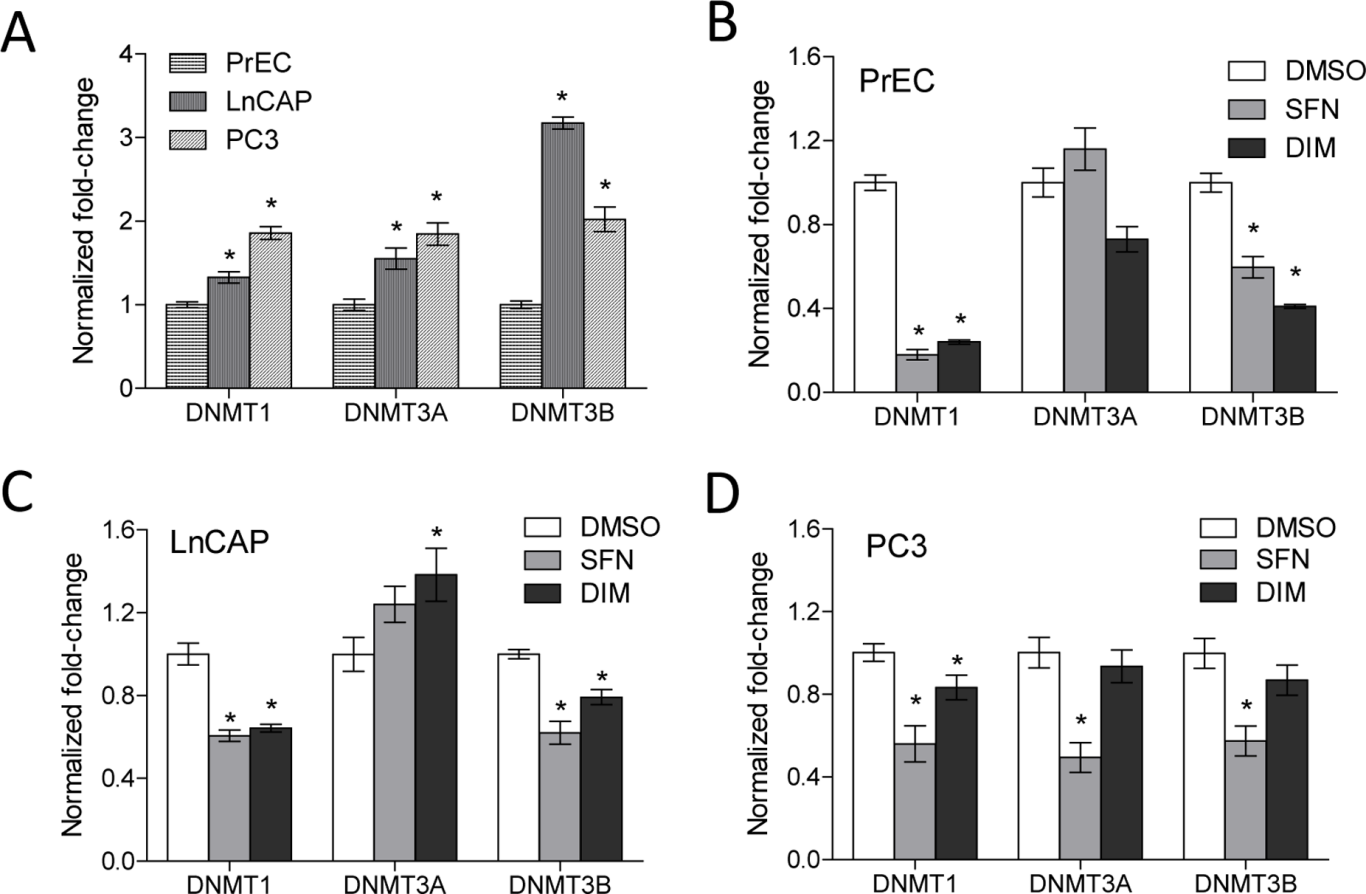
Increased DNA methyltransferase gene expression in prostate cancer cell lines was decreased with SFN or DIM treatments. (A) Gene expression of DNMT1, DNMT3A, and DNMT3B in untreated PrEC, LnCAP, and PC3 cells (n = 3–5 per group). Data represent mean normalized fold-change ± SEM compared to PrEC. (B–D) Effects of SFN and DIM on DNMT gene expression in PrEC cells (B), LnCAP cells (C), and PC3 cells (D). Cells were treated with vehicle control (DMSO), 15 µM SFN, or 15 µM DIM (n = 6 per group in B–D). DNMT1, DNMT3A, and DNMT3B gene expression were analyzed 48 h post-treatment. Data represent mean normalized fold-change ± SEM compared to DMSO. *p-value <0.05.

We assessed the effects of SFN and DIM on the expression of DNMT1, DNMT3A, and DNMT3B in normal prostate epithelial cells (PrEC), androgen-dependent (LnCAP) and androgen-independent (PC3) prostate cancer cells. LnCAP and PC3 cells had significantly higher baseline expression of DNMT1, DNMT3A, and DNMT3B compared to PrEC cells (Fig. 1A). In PrEC and LnCAP cells, both SFN and DIM treatments decreased DNMT1 and DNMT3B gene expression (Fig. 1B and 1C). In PC3 cells, SFN significantly decreased the expression of all three DNMTs examined, and DIM decreased the expression of DNMT1 (Fig. 1D). Based on this result, as well as our previous findings that SFN can mediate promoter de-methylation in prostate cancer cells [18], we performed a genome-wide survey to determine the global effects of SFN and DIM on promoter DNA methylation in PrEC, LnCAP, and PC3 cells.

**Figure 2:**
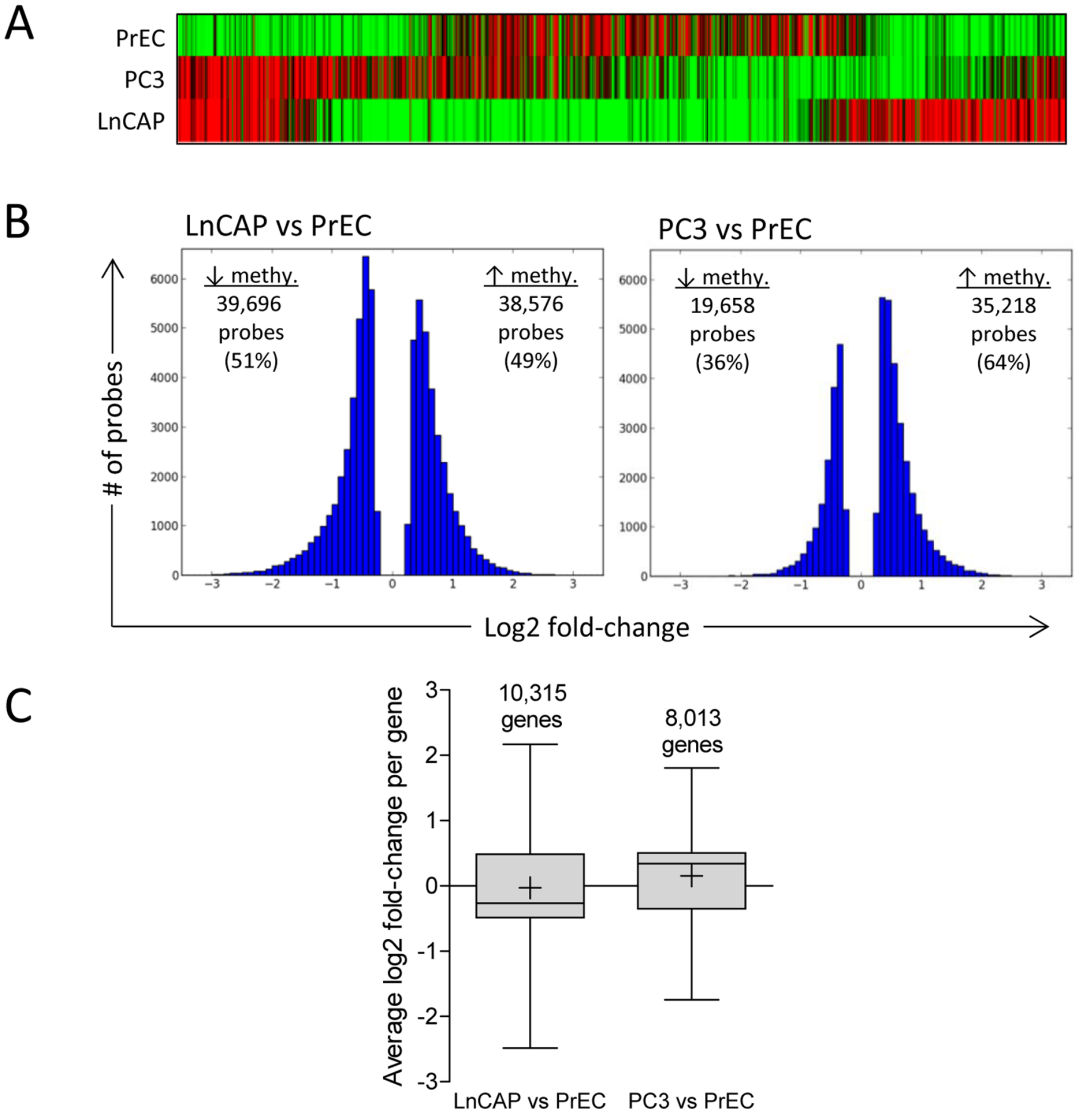
Genome-wide promoter methylation profiling comparing normal prostate epithelial cells and prostate cancer cells. (A) Hierarchical clustering analysis of probes with significant scaled log_2_ ratio in PrEC, LnCAP, and PC3 cells. Green and red bars represent individual probes with significant decreased and increased methylation, respectively, of MeDIP DNA samples relative to input DNA samples. Data represent the top 1,000 most significant probes within each cell line. (B) A comparison of methylation changes in LnCAP and PC3 cells relative to PrEC cells. Significant methylated probes in LnCAP and PC3 cells were compared to PrEC, and the distribution of probes with significant log2 fold-changes are shown. (C) Average methylation level in individual genes in LnCAP and PC3 cells compared to PrEC. Log2 fold-change per gene was determined by averaging the log2 fold-change of all differentially methylated probes assigned to each gene, and represented as box and whisker plots. Number above the bar denotes the number of genes in each group. Whiskers represent maximum and minimum values, and “+” represents mean value.

The methylation status of individual probes was first identified in each of the three cell lines by comparing MeDIP-enriched versus input samples (scaled log2 ratio). Hierarchical clustering analysis showed that PrEC, LnCAP, and PC3 had distinct methylation profiles (Fig. 2A). Next, differential methylation between prostate cancer cells and normal prostate epithelial cells were determined. Probes with significant scaled log_2_ ratio identified in LnCAP cells and PC3 cells were compared to those identified in PrEC cells via pairwise comparison to determine significant log2 fold-differences between prostate cancer cells and normal prostate epithelial cells (LnCAP versus PrEC, and PC3 versus PrEC). All subsequent analyses of cell line and/or treatment effects referred to significant methylation changes based on log2 fold-change comparisons. LnCAP cells and PC3 cells had 78,272 probes and 54,876 probes, respectively, that were significantly differentially methylated compared to normal PrEC cells (Fig. 2B). In PC3 cells, 64% of the probes had increased methylation, compared to 49% of the probes in LnCAP cells that had increased methylation relative to PrEC. Since the methylation profile of each gene was assessed by multiple probes, we determined the mean methylation level per gene by averaging the significant log2 fold-change of all probes assigned to individual genes. This represented 10,315 and 8,013 differentially methylated genes in LnCAP and PC3 cells, respectively, relative to PrEC cells (Fig. 2C). We observed that the majority of probes within each gene had similar methylation changes, where greater than 92% either had increased or decreased methylation. Only 7.4% of the probes in LnCAP cells and 4.2% of the probes in PC3 cells had mixed methylation profile within each gene (data not shown). Log2 fold-change per gene ranged from −3.322 to 2.893 in LnCAP cells, and −2.411 to 2.941 in PC3 cells. Functional annotation analyses indicated that genes with altered methylation profile in prostate cancer cells were enriched in genes that are dysregulated or involved in cancer progression, including GO categories associated with cell migration, cell adhesion, cell-cell signaling, as well as transcription regulation (data not shown). Select genes with differential methylation based on the NimbleGen methylation array were validated by pyrosequencing (Figure S1).

**Figure 3:**
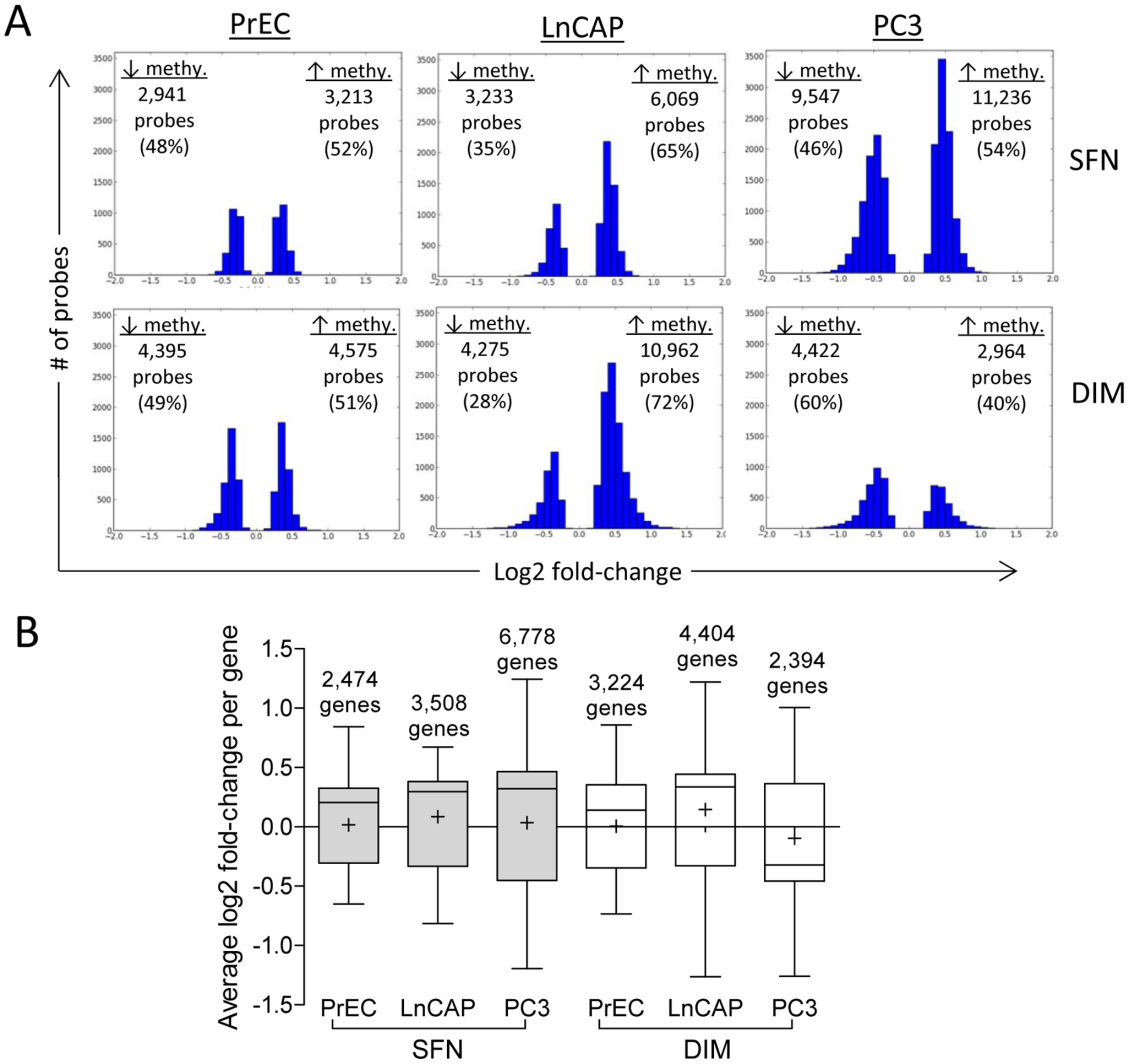
Genome-wide promoter methylation effects of SFN or DIM on normal prostate epithelial cells and prostate cancer cells. (A) Effects of SFN and DIM on the methylation profile in PrEC, LnCAP, and PC3 cells compared to vehicle control. In each of the three cell lines, significant methylated probes in SFN or DIM-treated groups were compared to their respective vehicle control to determine probe-specific log2 fold-change. The distribution of probes with significant log2 fold-change is shown. (B) Average methylation level in individual genes in SFN and DIM-treated PrEC, LnCAP, and PC3 cells compared to vehicle control. Log2 fold-change per gene was determined by averaging the log2 fold-change of all differentially methylated probes assigned to each gene, and represented as box and whisker plots. Number above the bar denotes the number of genes in each group. Whiskers represent maximum and minimum values, and “+” represents mean value.

We next examined the effects of SFN and DIM on the promoter methylation profiles in each prostate cell line. Log2 fold-change was compared between SFN or DIM treatments relative to DMSO vehicle control in PrEC, LnCAP, and PC3 cells. SFN treatments resulted in significant log2 fold-change in 6,154 probes in PrEC cells, 9,302 probes in LnCAP cells, and 20,783 probes in PC3 cells (Fig. 3A), representing 2,472, 3,508, and 6,778 differentially methylated genes, respectively (Fig. 3B). DIM treatments induced significant log2 fold-change in 8,970 probes in PrEC cells, 15,237 probes in LnCAP cells, and 7,386 probes in PC3 cells, representing 3,224, 4,404, and 2,394 differentially methylated genes, respectively (Fig. 3B). Individual probes within each gene had similar methylation changes, where greater than 95% of the probes either had increased or decreased methylation, and very few genes had mixed methylation profile (<5% of the probes in SFN-treated cells and <2.5% of the probes in DIM treated cells) (data not shown). The range of fold-change due to SFN and DIM treatments were between −1.380 to 1.243, and was narrower relative to those observed when comparing prostate cancer cells versus normal prostate epithelial cells (Fig. 2B). Distribution of the differentially methylated probes within the promoter was examined and showed no preferential bias to specific promoter regions (data not shown).

**Figure 4:**
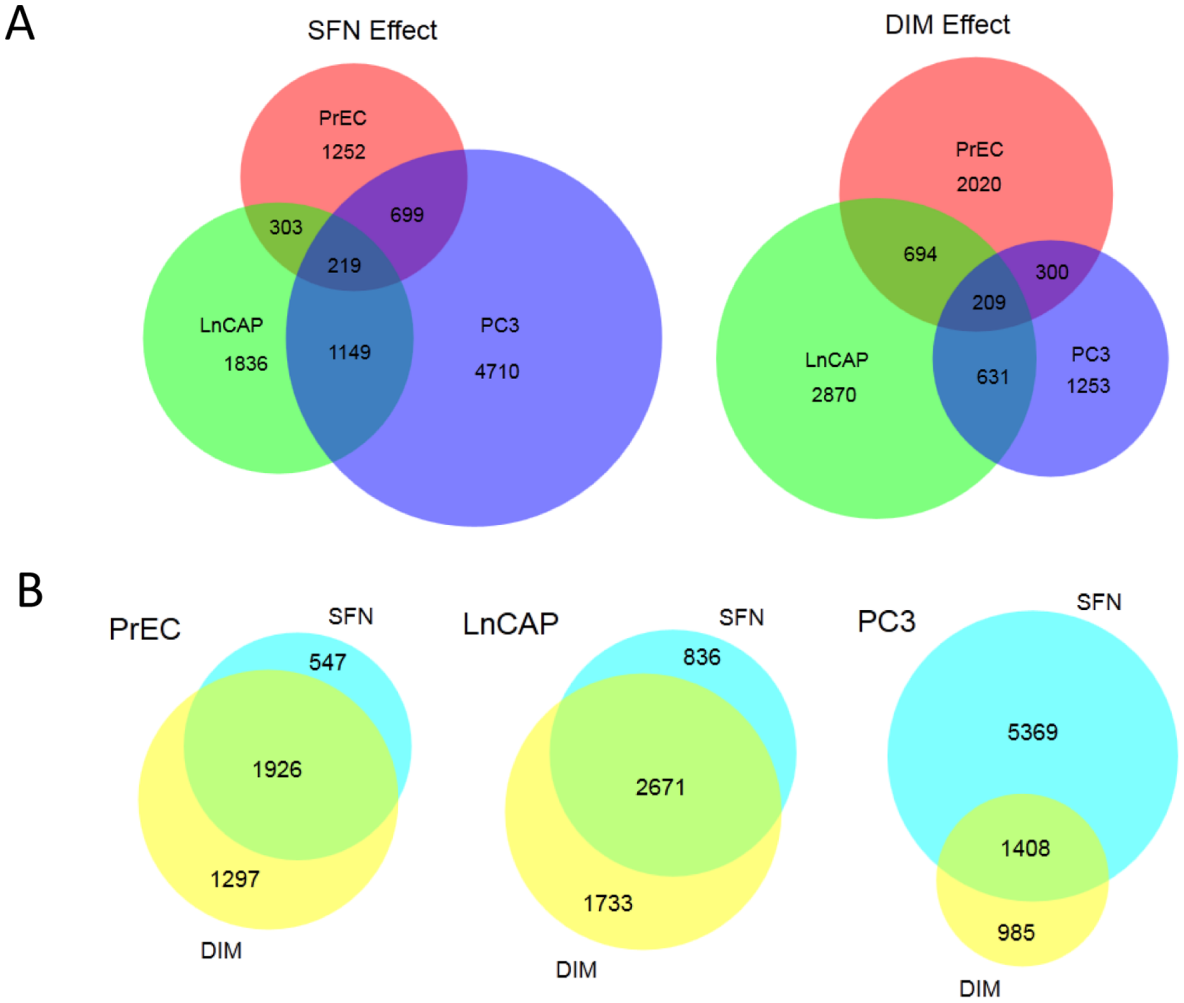
SFN and DIM had distinct methylation gene targets in normal prostate epithelial cells and prostate cancer cells but high level of gene targets overlap within a single cell line. (A) Venn diagrams showing the number of genes that were differentially methylated in PrEC, LnCAP, and PC3 cells upon treatments with SFN or DIM compared to vehicle control. (B) Venn diagrams showing the number of differentially methylated gene targets of SFN and DIM within each cell line.

**Figure 5:**
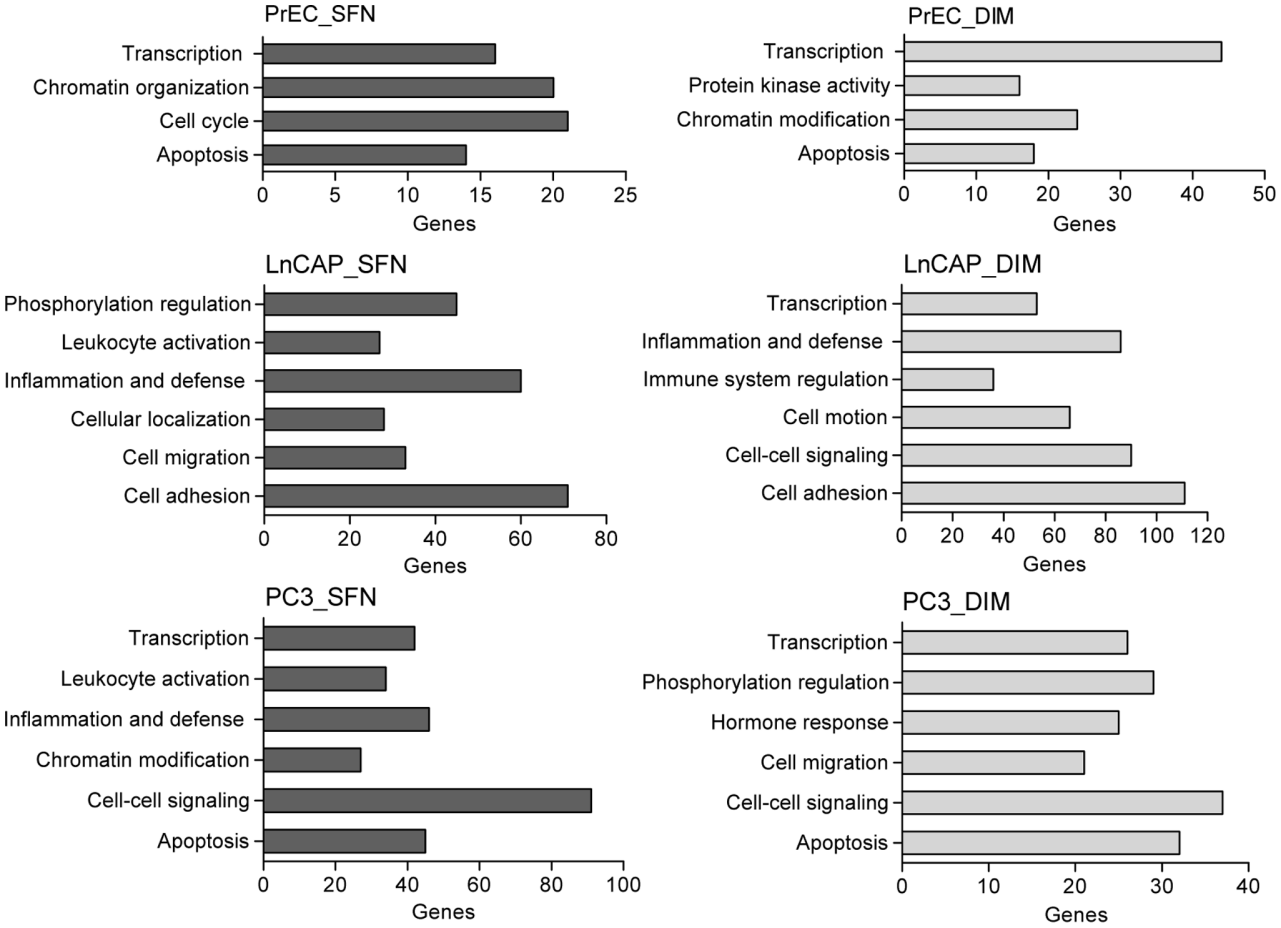
Biological functions of genes with promoter methylation affected by SFN or DIM treatments in normal prostate epithelial cells and prostate cancer cells. Functional annotation of differentially methylated gene targets of SFN and DIM in PrEC, LnCAP, and PC3 cells were examined and the number of genes involved in select biological processes and functions were shown. Data are expressed as the number of genes in each significantly enriched GO category.

Venn diagram analyses showed that SFN and DIM each altered methylation in distinct sets of genes in each of the cell lines. Comparison of the sets of genes altered by SFN or DIM showed that only a small number of genes (219 and 209 genes, respectively) were shared among all three cell lines, representing between 3% to 9% of differentially methylated genes within each cell line (Fig. 4A). Of this core set of genes, there were ∼30% overlap between SFN and DIM treatments. In contrast, there was a high degree of overlap of genes affected by both SFN and DIM treatments within PrEC and LnCAP cells (1,926 genes and 2,671 genes, respectively), and to a lesser extent PC3 cells (1,408 genes) (Fig. 4B). Similar results were obtained when we subdivided the datasets into genes with increased methylation or decreased methylation (data not shown). Functional annotation analyses showed different sets of enriched genes in normal prostate epithelial cells compared to prostate cancer cells, but similar sets of genes were enriched when comparing SFN and DIM treatment within each cell line (Fig. 5). In PrEC cells, genes involved in transcription, apoptosis and chromatin organization/modification were enriched with both SFN and DIM treatments. In LnCAP cells, two general categories of genes were enriched. The first involved genes associated with cell movement, including cell migration, adhesion, and localization. The second involved genes associated with immune response, including inflammation and defense, leukocyte activation and immune regulation. Functional annotation analysis in PC3 cells showed enriched gene categories shared similarity to both PrEC and LnCAP, including genes involved in transcription, apoptosis, cell migration, and immune response.

### SFN and DIM Reversed Cancer-associated DNA Methylation Alterations in LnCAP Cells

**Figure 6:**
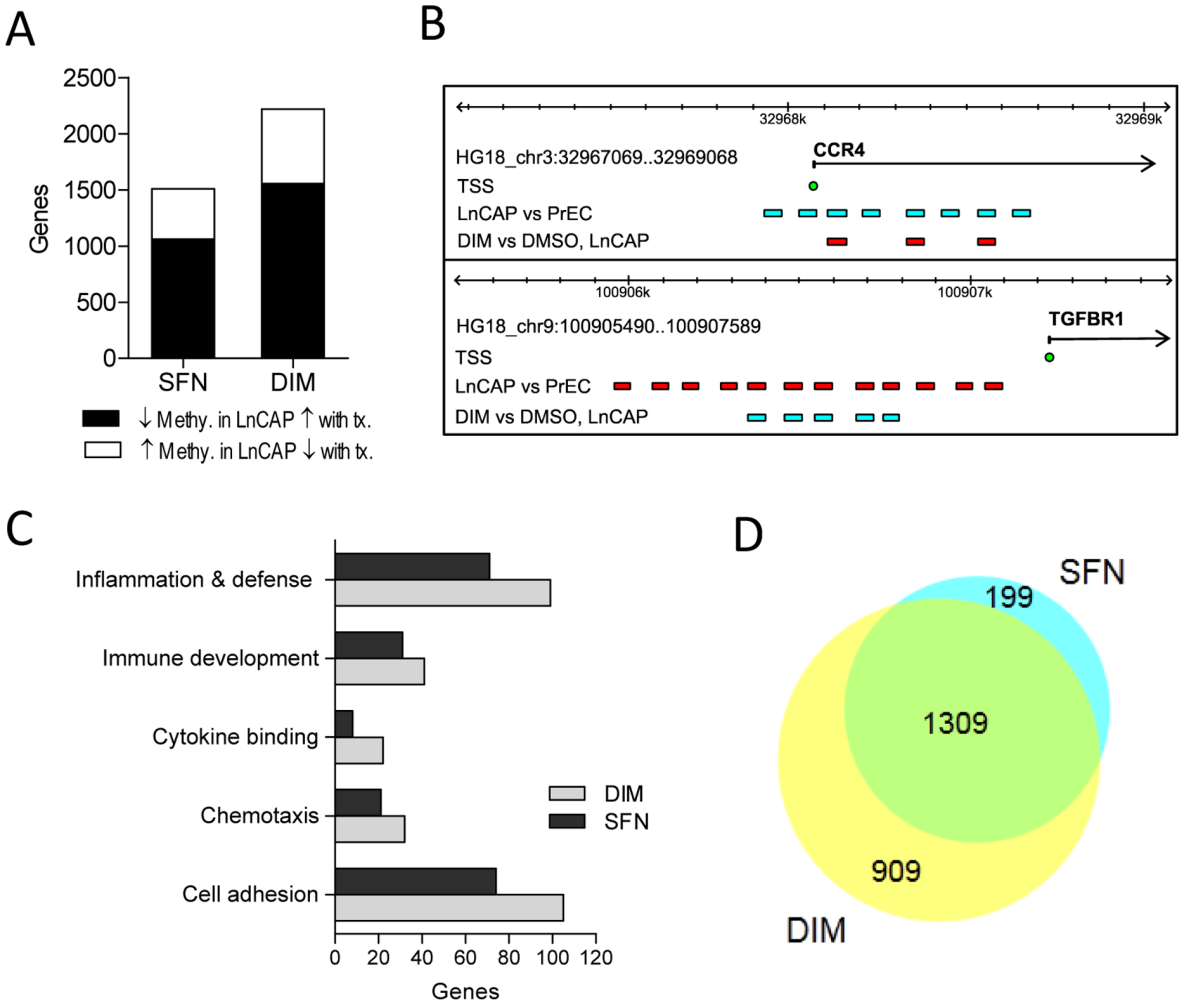
Functional annotation of genes with altered promoter methylation in LnCAP cells that were reversed with SFN and/or DIM treatments. (A) Number of differentially methylated genes in LnCAP cells relative to PrEC cells that were reversed with SFN or DIM treatments. Black bar represents genes that had decreased methylation in LnCAP cells that were increased with SFN/DIM treatments. White bar represents genes that had increased methylation in LnCAP cells that were decreased with SFN/DIM treatments. (B) Two gene examples, CCR4 and TGFBR1, with dysregulated methylation profiles in LnCAP cells that were reversed with SFN and/or DIM treatments. Colored bars represent the genomic position of DNA methylation probes that had decreased methylation (blue) or increase methylation (red) when comparing the probe-specific log2 fold-change in LnCAP cells versus PrEC, or DIM versus DMSO vehicle control in LnCAP cells. TSS (blue circle) represents transcription start site of each gene. (C) Functional annotation of differentially methylated gene targets in LnCAP cells that were reversed with SFN or DIM treatments. (D) Venn diagram showing the number of gene targets with promoter methylation that were dysregulated in LnCAP cells and reversed with SFN or DIM.

Next, given the more significant decrease in DNMT1 and DNMT3B with both SFN and DIM treatment in LnCAP cells, we chose to focus on characterizing a subset of genes which were differentially methylated in LnCAP cells relative to PrEC cells, and were reversed with SFN and/or DIM treatments. Of the 10,315 genes that had differential methylation in LnCAP cells (Fig. 2C), SFN and DIM treatments reversed the methylation profiles of 1,509 (14.6%) and 2,219 (21.5%) genes, respectively (Fig. 6A). These genes belonged to two categories: 1) genes that had increased promoter methylation in LnCAP cells relative to PrEC cells, and reduced methylation upon SFN and/or DIM treatment, and 2) genes that had decreased promoter methylation in LnCAP cells relative to PrEC cells, and increased methylation upon SFN and/or DIM treatment. An example of a gene that belongs to each category, C–C chemokine receptor type 4 (CCR4) and transforming growth factor-β1 receptor type I (TGFBR1), was shown in Fig. 6B. Functional annotation analyses showed GO enrichment for many genes known to be dysregulated or are highly involved in cancer progression. This dataset included genes involved in cell adhesion and chemotaxis, as well as immune-related genes involved in inflammation and defense, cytokine binding, and immune development (Fig. 6C). Venn diagram comparison showed that 86% of the genes in the SFN gene list (1309 out of 1509) were shared with the DIM gene list (Fig. 6D). Since the majority of genes affected by SFN were included in the DIM gene list, subsequent gene expression analyses focused on select genes where DIM treatment reversed the dysregulated methylation profile in LnCAP cells.

**Figure 7:**
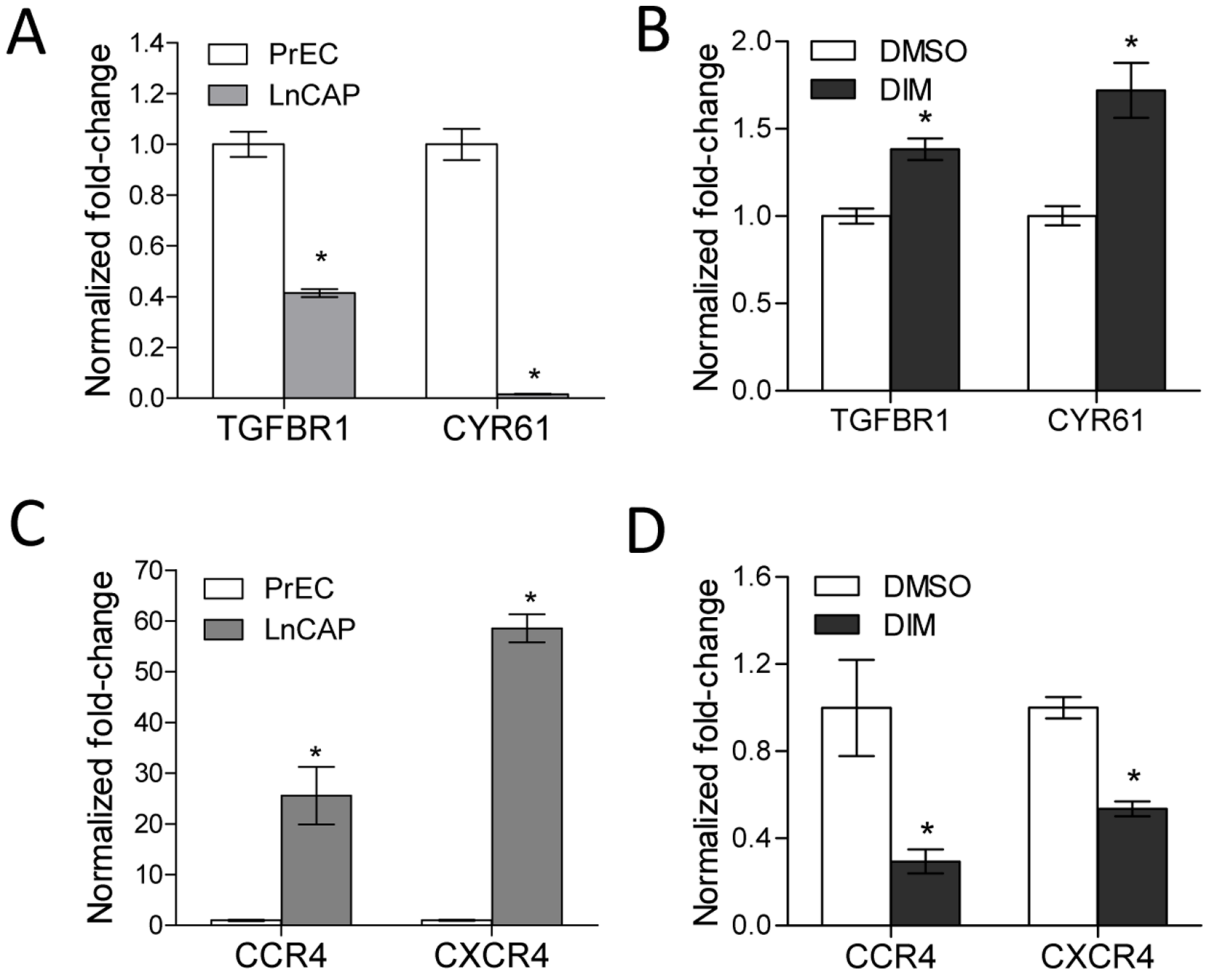
DIM treatment resulted in both the re-expression of hypermethylated genes, as well as reduced expression of hypomethylated genes. (A) Relative gene expression of TGFBR1 and CYR61 in PrEC and LnCAP cells (n = 3 for PrEC, n = 7 for LnCAP). (B) Effect of DIM on TGFBR1 and CYR61 gene expression in LnCAP cells (n = 7). (C) Relative gene expression of CCR4 and CXCR4 in PrEC and LnCAP cells (n = 3 for PrEC, n = 7 for LnCAP). (D) Effect of DIM on CCR4 and CXCR4 gene expression in LnCAP cells (n = 7). Data represent mean normalized fold-change ± SEM compared to PrEC in (A) and (C), or mean normalized fold-change ± SEM compared to DMSO vehicle control in (B) and (D). *p-value <0.05.

Four genes (TGFBR1, cysteine-rich angiogenic inducer 61 (CYR61), CCR4, and C-X-C chemokine receptor type 4 (CXCR4)) were selected to further examine the relationship between alteration in promoter methylation profile and gene expression in LnCAP cells. The promoter methylation profiles of each of these four genes were altered in LnCAP cells compared to PrEC cells, and DIM treatment reversed the cancer-associated methylation profiles. The expression of each of the four candidate genes have been reported to be dysregulated in cancer, and correlated with cancer progression. A loss of expression of TGFBR1 and CYR61 has been associated with prostate cancer progression and/or recurrence among many other cancer types [31]–[34]. Data from our methylation array showed that the promoter regions of TGFBR1 and CYR61 were hypermethylated in LnCAP cells (Fig. 6B and data not shown). This correlated with a significantly decreased gene expression in LnCAP cells relative to PrEC cells (Fig. 7A). DIM treatment resulted in decreased promoter methylation when compared to cells treated with vehicle control (Fig. 6B and data not shown), and was associated with significantly increased in TGFBR1 and CYR61 gene expression (Fig. 7B). Increased gene expression were likely due to reduced promoter methylation, as LnCAP cells treated with DNA demethylation agent, AZA, similarly resulted in increased TGFBR1 and CYR61 expression (Figure S2). In contrast, overexpression of the chemokine receptors CCR4 and CXCR4 have been associated with poor prognosis and the promotion of a variety of cancers including prostate cancer [35]–[38]. Data from our methylation array showed that the promoter regions of CCR4 and CXCR4 were hypomethylated in LnCAP cells (Fig. 6B and data not shown), and correlated with significantly increased gene expression in LnCAP cells relative to PrEC cells (Fig. 7C). DIM treatment resulted in increased promoter methylation when compared to cells treated with vehicle control (Fig. 6B and data not shown), and was associated with a significant decrease in CCR4 and CXCR4 gene expression (Fig. 7D).

**Figure 8:**
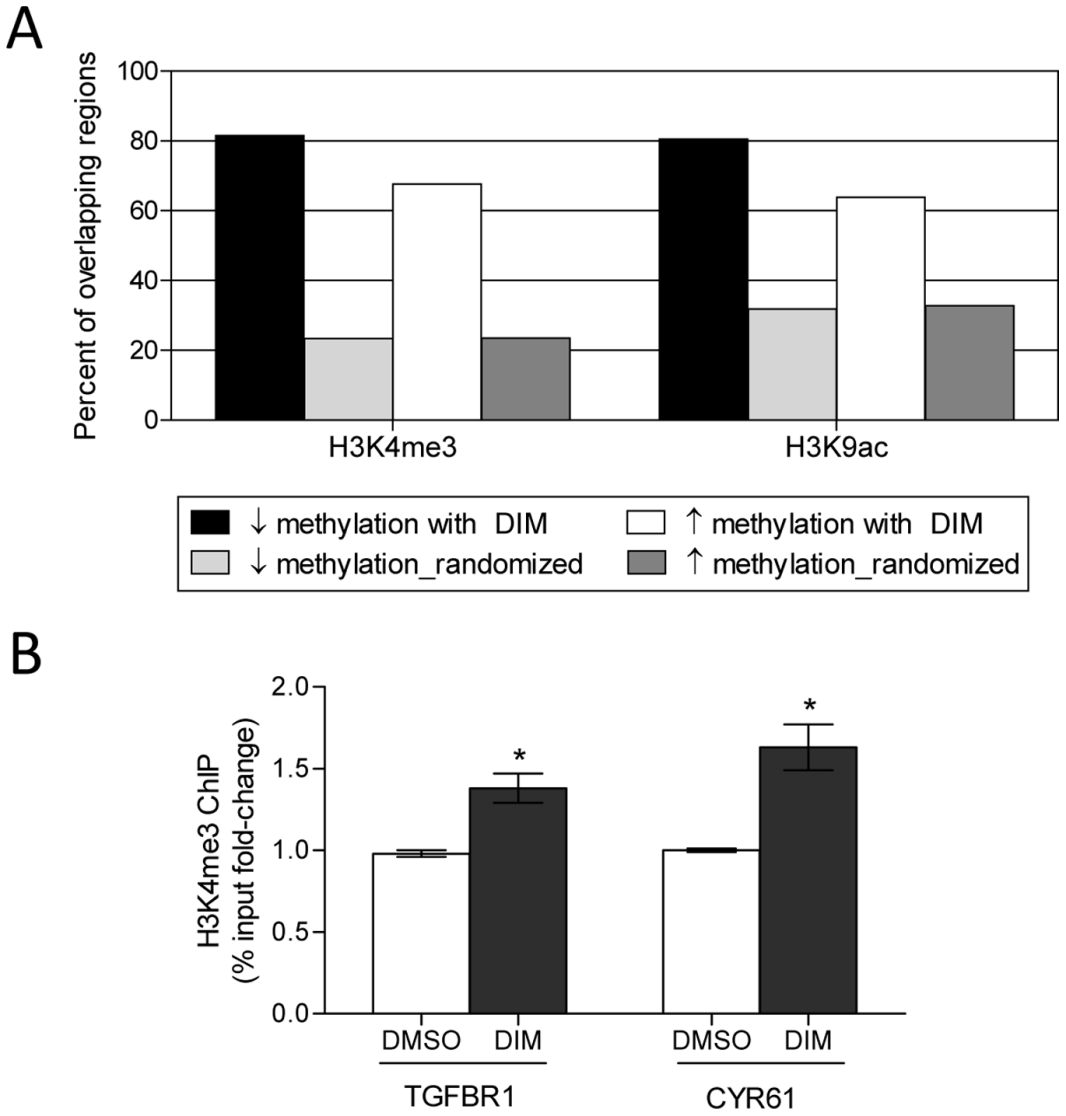
Comparison of the overlap of DNA methylation probes with epigenetic marks showed the outcome of DIM-mediated alteration in DNA methylation may be influenced by local histone modifications. (A) The genomic region coordinates of DNA methylation probes that had decreased methylation (black bar) or increased methylation (white bar) in DIM-treated LnCAP cells were uploaded into EpiExplorer to determine potential overlap with various histone modifications from the ENCODE database. A randomized control set was generated by reshuffling the genomic positions of each dataset (light and dark gray bars for decreased methylation and increased methylation, respectively). Data summarize the percent overlap of DNA methylation probes and their respective randomized control to histone methylation mark H3K4me3, and histone acetylation marks H3K9ac. (B) ChIP assays for H3K4me3 at the TGFBR1 and CYR61 promoters in LnCAP cells after vehicle (DMSO) or 15 µM DIM treatments for 48 h. ChIP-qPCR data were expressed as % input. Results represent the mean % input fold-change ± SEM compared to DMSO vehicle control of two independent experiments. *p-value <0.05.

Our data suggested that DIM treatment can result in both the re-expression of DNA methylation silenced genes by decreasing promoter methylation, as well as reducing the expression of hypomethylated genes by increasing promoter DNA methylation. Since local chromatin histone modifications play an important role in establishing DNA methylation patterns, we explored the possibility that the opposing outcomes of DIM-mediated alteration in DNA methylation in our dataset were in part influenced by local histone marks. We used EpiExplorer to examine the potential association of differentially methylated regions with various epigenetic marks [27]. Specifically, the genomic region coordinates of DNA methylation probes that had significantly decreased methylation (4,275 probes), or increased methylation (10,962 probes) in DIM-treated LnCAP cells were uploaded as separate datasets into EpiExplorer and annotated with genomic attributes based on ENCODE data [39]. This analysis enabled us to determine if there were different epigenetic attributes associated with the two datasets that may potentially explain the difference in methylation outcome. A comparison of the overlap of DNA methylation probes with specific histone marks showed enrichment of DNA methylation probes with histone methylation and histone acetylation marks associated with active promoter and open chromatin conformation, including H3K4me3 and H3K9ac (Fig. 8A). Methylation probes and ENCODE histone datasets were exported into Genomic HyperBrowser for statistical analyses [29], [30]. Enrichment and overlap of DNA methylation probes with both H3K4me3 and H3K9ac histone marks were significantly above those expected by chance when compared to randomized control (p<0.001), suggesting the observed enrichments were of potential biological relevance. Interestingly, probes associated with DIM-mediated decreased DNA methylation had a significantly higher degree of overlap with H3K4me3 and H3K9ac (81.5%, and 80.5%, respectively) when compared to probes associated with DIM-mediated increased DNA methylation, which had less overlap with H3K4me3 and H3K9ac (67.6% and 63.9%, respectively) (Two-sample proportion t-test, p<0.001). Neither category of probes showed enrichment for histone marks associated with repressed/silenced chromatin, including H3K9me3 and H3K27me3, when compared to their respective randomized control (data not shown). Correlation between DIM-mediated decrease in DNA methylation and increase association with H3K4me3 histone modification was further confirmed experimentally. Specifically, the association of H3K4me3 with the promoter regions of TGFBR1 and CYR61 in LnCAP cells treated with vehicle control or DIM was analyzed by ChIP assays. A significant increase in H3K4me3 at both TGFBR1 and CYR61 promoters was observed in LnCAP cells after DIM treatment when compared to vehicle control (Fig. 8B).

## Discussion

Epigenetic changes, including aberrant DNA methylation, result in altered gene expression and play an important role in carcinogenesis. Phytochemicals such as SFN and DIM can modulate gene expression via epigenetic mechanisms, and are promising chemopreventive agents for the treatment of prostate cancer. In cancer cells, SFN modulated promoter methylation and expression of cyclin D2 and hTERT via the inhibition of DNMT [18], [20]. However, it was unclear the scope of SFN’s effects on the methylation of other gene targets within the genome, and whether SFN has differential methylation effects on normal prostate epithelial cells versus prostate cancer cells. While DIM has similar chemopreventive properties as SFN, the ability of DIM to modulate DNA methylation has not been investigated. We provided for the first time, evidence of widespread changes in promoter methylation patterns in both normal and cancerous prostate cells in response to SFN or DIM treatments. DNA methylation targets were largely distinct, depending on cell line. We further showed that SFN and DIM reversed many of the cancer-associated DNA methylation alterations, including aberrantly methylated genes that are dysregulated or are highly involved in cancer progression. Our EpiExplorer and ChIP analyses suggested that the differential DNA methylation outcome mediated by DIM treatment may potentially be influenced by local histone modifications, which warrants future testing and confirmation. Overall, our results suggest that both SFN and DIM are epigenetic modulators that have broad effects in altering aberrant DNA methylation patterns, but may have differing targets depending on the stage of cancer. Together these studies help reveal important novel mechanisms for SFN and DIM in prostate cancer chemoprevention.

Dysregulation and reprogramming of the epigenome contribute to cancer initiation and progression. Prostate cancer is associated with global and site-specific DNA hypomethylation, as well as site-specific hypermethylation. While DNA hypomethylation leads to genomic instability and is associated with prostate cancer disease progression [40], [41], to date the majority of aberrantly methylated genes identified in prostate cancer are hypermethylated and relatively few promoters have been reported to be hypomethylated [42]. A recent review reported ∼80 genes in prostate cancer with promoter hypermethylation [43]. Using the NimbleGen methylation array that surveyed 22,532 promoters, we observed a much larger set of genes with altered promoter methylation in the prostate cancer cell lines LnCAP and PC3 when compared to normal PrEC cells (Fig. 2). Our data also showed that while 64% of the promoters in PC3 cells had increased methylation, LnCAP cells did not show a bias towards hypermethylated promoters, as only 49% of the genes had increased methylation compared to PrEC cells. As expected, GO analysis revealed that genes that have altered DNA methylation were associated with cancer progression, including genes involved in cell migration, cell adhesion, cell-cell signaling, as well as transcription regulation, reaffirming the notion that aberrant DNA methylation contributes to tumorigenesis. Results from our genome-wide methylation profiling provided a comprehensive survey of promoter methylation in prostate cancer cells which allow us to identify previously unknown gene targets with altered promoter methylation.

Overexpression of DNMT is associated with many cancers including prostate cancer (Fig. 1A), and contributes to epigenetic silencing of tumor suppressor genes. DNMT inhibitors represent a promising class of epigenetic modulator currently being tested in cancer therapy. Bioactive food components have been shown to have DMNT inhibitory properties that may influence DNA methylation patterns. We showed that SFN and DIM were both effective in inhibiting DNMT gene expression (Fig. 1). Reduced DNMT expression was expected to result in an overall decrease in promoter methylation. Instead, our data suggested that SFN and DIM had broad and complex genome-wide effects on DNA methylation patterns, affecting both normal and cancerous prostate cell lines (Fig. 3). Notably, while SFN and DIM shared similar gene targets within a single cell line, distinct gene targets were differentially methylated in PrEC, LnCAP, and PC3 cells (Fig. 4 and 5). Our results suggest that the effects of SFN and DIM on DNA methylation could not simply be explained by their DNMT inhibitory activities, and additional factors were likely involved in dictating the outcome of DNA methylation. We were specifically interested in genes with altered promoter methylation profile in prostate cancer cells that were reversed with SFN and/or DIM treatments. This group included genes associated with biological processes highly involved in cancer progression such as cell adhesion, chemotaxis, and inflammation (Fig. 6). In particular, the role of chemokines in cancer progression, including prostate cancer, has been well established. This includes chemokine-mediated modulation of cell proliferation, angiogenesis, chemotaxis and recruitment of leukocytes, activation of proinflammatory mediators, and augmentation of tumor invasion and metastasis [44], [45]. Notably, SFN and DIM exert anti-cancer effects by suppressing many of these chemokine-related processes [46]–[49]. Our data suggest that one of the mechanisms by which SFN and DIM exert these chemoprevention effects may be via the modulation and reversal of aberrant promoter DNA methylation in chemokine-related gene targets in cancer cells.

The regulation of DNA methylation and gene expression is influenced by complex interactions between DNA methylation and histone modifications [3], [50], [51]. Histone modifying enzymes can affect the recruitment, stability, and function of DNMT, as well as direct DNA methylation to specific genomic targets. DNMT in turn plays a role in recruiting HDAC and methyl-CpG binding protein that affects chromatin structure. We showed that SFN and DIM could mediate both gene- and cell line-specific increase and decrease promoter methylation, and we reasoned that this may in part be dependent upon the presence or absence of other chromatin-associated proteins, as well as the context of local histone modifications that collectively influence local chromatin structure and accessibility. Indeed, DNA methylation probes in DIM-treated LnCAP cells that had decreased methylation were enriched for histone marks associated with open chromatin (Fig. 8). However, we also anticipated that DNA methylation probes with increased methylation due to DIM treatments would be more frequently associated with repressive histone marks. This was not observed in our bioinformatic analyses, and could be attributed to the limitations in using public reference data, as very little is currently known regarding the impact of DIM (and SFN) on the epigenome. In addition, DIM (and SFN) may potentially have selective effects on specific histone modifications that will need to be determined. In this paper we have chosen to focus our analysis on DIM-treated LnCAP cells to determine the relationship between DNA methylation alterations, gene expression, and the potential association with specific histone marks. Future work will include further characterization of the effects of both SFN and DIM on all three prostate cell lines. The influence of SFN and DIM on DNA methylation patterns is likely mediated by their effects on multiple epigenetic processes. Additional experiments will be needed to fully define the epigenetic effects of SFN and DIM. Overall, we demonstrated that SFN and DIM may act as dietary chemoprevention agent for the treatment of prostate cancer via their ability to modulate DNA methylation and regulate gene expression. A better understanding of the epigenetic modulatory mechanisms of SFN and DIM on gene regulation will be an important area for future studies.

## Supporting Information

Figure S1 **Validation of NimbleGen DNA methylation array data by pyrosequencing.** Promoter DNA methylation status of select genes with differential methylation in LnCAP cells (black bars) compared PrEC cells (white bars) based on NimbleGen methylation array data were confirmed by pyrosequencing. (A) Promoter methylation of CCR4, IL10, and ITGAL. All three genes had reduced methylation in LnCAP compared to PrEC based on methylation array data. (B) Promoter methylation of SMAD3, TGFBR1, and WNT5A. All three genes had increased methylation in LnCAP compared to PrEC based on methylation array data. Data represent mean percent methylation by pyrosequencing ± SEM (n = 3 per cell line). *p-value <0.05 compared to PrEC cells. Methylation array fold-change (LnCAP/PrEC) for each of the six genes were listed for reference.
(PDF)

Figure S2 **AZA treatment resulted in the re-expression of methylation-silenced genes in LnCAP cells.** Relative gene expression of TGFBR1 and CYR61 in LnCAP cells left untreated (UT), or treated with 5 µM AZA for 48 h (n = 5 per group). Data represent mean normalized fold-change ± SEM compared to untreated control. *p-value <0.05.
(PDF)

Table S1 **Pyrosequencing primers.** Pyrosequencing PCR and sequencing primers for select differentially methylated genes.
(PDF)
