# Supplementary material for: Effects of Sulforaphane and 3,3′-Diindolylmethane on Genome-Wide Promoter Methylation in Normal Prostate Epithelial Cells and Prostate Cancer Cells
Source: PLoS One. 2014 Jan 22;9(1):e86787. doi: 10.1371/journal.pone.0086787 (PMC3899342; doi:10.1371/journal.pone.0086787)
Supplement: Figure S1 — Validation of NimbleGen DNA methylation array data by pyrosequencing. Promoter DNA methylation status of select genes with differential methylation in LnCAP cells (black bars) compared PrEC cells (white bars) based on NimbleGen methylation array data were confirmed by pyrosequencing. (A) Promoter methylation of CCR4, IL10, and ITGAL. All three genes had reduced methylation in LnCAP compared to PrEC based on methylation array data. (B) Promoter methylation of SMAD3, TGFBR1, and WNT5A. All three genes had increased methylation in LnCAP compared to PrEC based on methylation array data. Data represent mean percent methylation by pyrosequencing ± SEM (n = 3 per cell line). *p-value <0.05 compared to PrEC cells. Methylation array fold-change (LnCAP/PrEC) for each of the six genes were listed for reference. (PDF) [file pone.0086787.s001.pdf]

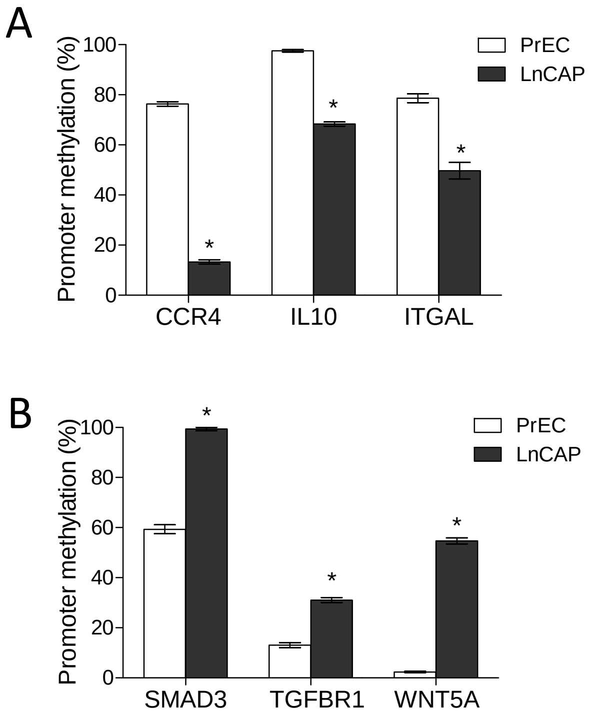

NimbleGen methylation array
LnCAP/PrEC fold-change

CCR4 0.69
IL10 0.70
ITGAL 0.49

NimbleGen methylation array
LnCAP/PrEC fold-change

SMAD3 3.39
TGFBR1 2.83
WNT5A 2.66

Figure S1. Validation of NimbleGen DNA methylation array data by pyrosequencing. Promoter DNA methylation status of select genes with differential methylation in LnCAP cells (black bars) compared PrEC cells (white bars) based on NimbleGen methylation array data were confirmed by pyrosequencing. (A) Promoter methylation of CCR4, IL10, and ITGAL. All three genes had reduced methylation in LnCAP compared to PrEC based on methylation array data. (B) Promoter methylation of SMAD3, TGFBR1, and WNT5A. All three genes had increased methylation in LnCAP compared to PrEC based on methylation array data. Data represent mean percent methylation by pyrosequencing ± SEM (n=3 per cell line). *p-value < 0.05 compared to PrEC cells. Methylation array fold-change (LnCAP/PrEC) for each of the six genes were listed for reference.
