# Supplementary material for: Effects of Sulforaphane and 3,3′-Diindolylmethane on Genome-Wide Promoter Methylation in Normal Prostate Epithelial Cells and Prostate Cancer Cells
Source: PLoS One. 2014 Jan 22;9(1):e86787. doi: 10.1371/journal.pone.0086787 (PMC3899342; doi:10.1371/journal.pone.0086787)
Supplement: Figure S2 — AZA treatment resulted in the re-expression of methylation-silenced genes in LnCAP cells. Relative gene expression of TGFBR1 and CYR61 in LnCAP cells left untreated (UT), or treated with 5 µM AZA for 48 h (n = 5 per group). Data represent mean normalized fold-change ± SEM compared to untreated control. *p-value <0.05. (PDF) [file pone.0086787.s002.pdf]

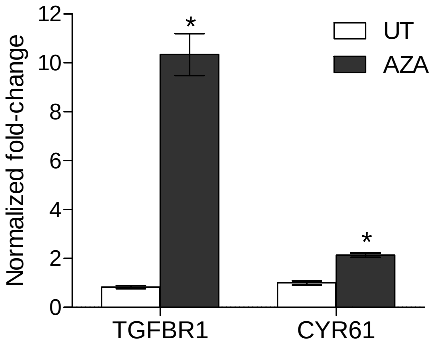

Figure S2. AZA treatment resulted in the re-expression of methylation-silenced genes in LnCAP cells. Relative gene expression of TGFBR1 and CYR61 in LnCAP cells left untreated (UT), or treated with 5μM AZA for 48h (n=5 per group). Data represent mean normalized fold-change ± SEM compared to untreated control. *p-value < 0.05.
