# Supplementary material for: Effects of Sulforaphane and 3,3′-Diindolylmethane on Genome-Wide Promoter Methylation in Normal Prostate Epithelial Cells and Prostate Cancer Cells
Source: PLoS One. 2014 Jan 22;9(1):e86787. doi: 10.1371/journal.pone.0086787 (PMC3899342; doi:10.1371/journal.pone.0086787)
Supplement: Table S1 — Pyrosequencing primers. Pyrosequencing PCR and sequencing primers for select differentially methylated genes. (PDF) [file pone.0086787.s003.pdf]

Table S1: Pyrosequencing primers

Gene	Forward primer	Reverse primer (Biotinylated)	Sequencing primer
CCR4	TGAATTTAGTATAGTTGGGTGAGGG	AATCTATAAAACAATACCAAACTTACTCA	GGTGAGGGAGATAATT
IL10	TTTTGGGGAGAATAGTTGTTTTG	CCCAATTATTTCTCAATCCCATTATAT	GGGAGAATAGTTGTTTTGT
ITGAL	TGTAAGAGGTTAAAGGGTATGATTAT	TCCCCTACCTCCTCCCTTA	TTTGTTTAGGTTGTTAGTAAAT
SMAD3	GTGGGGTATTTGTTATTGAGAAGTA	CATACCTCAATCTCCTCATCTATAA	TTTTTTTGGGTTTATTTTATTAGTA
TGFBR1	AGGGAGGAGGGAGGTTATTT	ACAACCCCAAACCCCTACATCCA	TTGGGTTTGGGGTGA
WNT5A	AGTTGGGATGAGTTTAGGAATGGA	CCCACATTTAAAATTAAAAAACCC	TAGGAATGGAGGGGG
